# Folate Receptor-Alpha Targeted 7x19 CAR-*γδ*T Suppressed Triple-Negative Breast Cancer Xenograft Model in Mice

**DOI:** 10.1155/2022/2112898

**Published:** 2022-03-07

**Authors:** Xueshuai Ye, Xinna Deng, Junye Wen, Yang Li, Mengya Zhang, Ziqi Cai, Guan Liu, Hezhi Wang, Jianhui Cai

**Affiliations:** ^1^Department of Surgery, Hebei Medical University, Shijiazhuang 050051, Hebei Province, China; ^2^Department of Oncology & Surgery, Hebei General Hospital, Shijiazhuang 050051, Hebei Province, China; ^3^Hebei Cell Therapy Technology Innovation Center, HOFOY Medicine Hebei Co., LTD, Shijiazhuang 050051, Hebei Province, China; ^4^Department of Liver Surgery & Transplantation, Liver Cancer Institute, Zhongshan Hospital of Fudan University, Shanghai City 200032, China

## Abstract

**Background:**

Triple-negative breast cancer (TNBC) is the worst prognosis subtype of breast cancer due to lack of specific targets. Recent studies have shown that immunotherapy may solve that problem by targeting folate receptor-alpha (FR*α*).

**Methods:**

Gene modified *γδ* T cells were manufactured to express FRa specific chimeric antigen receptor (FRa CAR) and secrete interleukin-7 (IL-7) and chemokine C–C motif ligand 19 (CCL19). CAR-*γδ*T cells that secrete IL-7 and CCL19 (7 × 19 CAR-*γδ*T) were evaluated for their antitumor activity both in vitro and in vivo.

**Results:**

7 × 19 CAR-*γδ*T showed remarkable antitumor activity in vitro. Combined with PBMC, 7 × 19 CAR-*γδ*T inhibited TNBC xenograft model growth superiorly compared with single-application or conventional CAR-*γδ*T cells. Histopathological analyses showed increased DC or T cells infiltration to tumor tissues.

**Conclusion:**

Taken together, our results showed that 7 × 19 CAR-*γδ*T have remarkable anti-TNBC tumor activity and showed a broad application prospect in the treatment of incurable TNBC patients.

## 1. Introduction

Triple-negative breast cancer (TNBC) is a kind of deadly disease, and there are nearly 15% of invasive breast cancer are TNBC [1]. The expression of human epidermal growth factor receptor 2 (HER2), estrogen receptor (ER), and progesterone receptor (PR) is negative in TNBC patients. Thus, endocrinotherapy and HER-2 targeted therapy were ineffective when treating TNBC, which necessitates novel treatment methods for TNBC patients. Chimeric antigen receptor (CAR) T cell had shown brilliant antitumor ability in hematologic malignancy by targeting the certain antigen such as CD19 and CD20 [2]. Second generation of CAR-T cells has an intracellular signaling domain consisting of CD3 *ζ* and one costimulatory molecule, such as CD28 or CD137, while the third generation CAR-T cells has nearly the same structure besides two costimulatory molecules. However, the treatment effect of second and third generation CAR-T cells is still controversial [3]. There are still many obstacles to overcome in the application of CAR-T cell therapy fighting against solid tumor. Unlike most hematologic malignancy tumor cells dispersed in the peripheral vascular or lymphatic system, solid tumor cells are the absence of ideal targets and usually grew as a lump. Aggregation and growth of tumor cells formed an immune impressive tumor microenvironment (TME), which is low oxygen, low pH, high metabolic waste, high immune-suppressive cytokines, and the upregulation of immune-checkpoint, such as PD-L1 and CTLA-4; those factor made the immune cells can hardly recognize and lyse the cancer cell [4, 5].

Besides, autologous T cells engineered to express a CAR also limit the application of CAR-T as an “off the shelf” drug to some extent. The ideal universal donor-derived T cell resources are *γδ*T cells or HLA and MHC depleted *αβ* T cells. Those *αβ* T cells can be produced by using gene editing tools such as TALEN [6], ZFN, and CRISPR/CAS9 to disrupt the endogenous TCR and HLA class I (HLA-I) [7, 8], while the *γδ*T cells can be safely applied in the allogeneic graft and *γδ*T cells can secrete CXCL13 naturally, which regulated the migration of B cells [9].

CCL19 is a chemoattraction for T cells and DCs [10, 11]. DCs plays an essential role in cell immunity through antigen presentation, T cells activation, and multiple cytokine releases, which mean CCL19 may break the TME by attracted DCs infiltration. IL-7 is known to enhance the proliferation and survival of T cells [12–14]. On the one hand, IL-7 can protect the T cells form inhibitory of TME. Previous reports have suggested that IL-7 signaling can prevent or restore T-cell exhaustion by means of multiple mechanisms, including downregulating PD-1 expression and Treg cell's function [15]. By introducing CCL19 and IL-7 into the conventional CAR structure, Koji Tamada et al. found that the 7 × 19 CAR-T can inhibit the growth of multiple types of solid tumors [16]. This research suggested that 7 × 19 CAR structure-based cell therapies may also bring hope for the TNBC treatment.

Several potential antigen targets, such as TEM8/ANTXR1 [17], NKG2D [18], MUC1 [19], and AXL, have been researched in TNBC. Folate receptor-alpha (FR*α*) is highly expressed in nonmucinous tumors of epithelial origin including ovarian, breast, and lung cancers and expressed at low levels on the apical surface of a subset of polarized epithelial cells, including the parotid, kidney, lung, thyroid, and breast. Specific overexpression of FR*α* is present in certain malignancies, including TNBC [20]. With low coordinate expression in normal tissue, FR*α* is an attractive target. However, the conventional second generation CAR-T targeting FRa did not inhibit tumor growth in those TNBC moderated expression model [21].

In the present study, we constructed a FRa specific mov19 scFv-based 7 × 19 CAR containing a fused CD28/CD3*ζ* endodomain and two 2A peptide to separate CCL19 and IL-7. Our results demonstrated that the 7 × 19 CAR-*γδ*T cell can strongly suppress TNBC tumor in vivo through various mechanisms.

## 2. Methods

### 2.1. Cell Lines

HEK-293T cell line was purchased from American Type Culture Collection (ATCC, Manassas, VA, USA) and used for lentivirus packaging and maintained in DMEM medium supplemented with 10% FBS (Gibco, Life Technologies, Rockville, MD, USA), 100 U/mL penicillin, 100 mg/mL streptomycin sulfate (Life Technologies, Rockville, MD, USA), and 10 mmol/L HEPES. Human breast cancer line MCF-7, human TNBC cell line MDA-MB-231, MDA-MB-436, and MDA-MB-468 were used to determine the biological activity of immune cells. Human ovarian cancer cell line C30 was used as negative control. All the tumor cell lines were maintained in L15 medium supplemented with 10% FBS, 100 U/mL penicillin, and 100 mg/mL streptomycin sulfate.

### 2.2. CAR Construction

**Figure 1:**
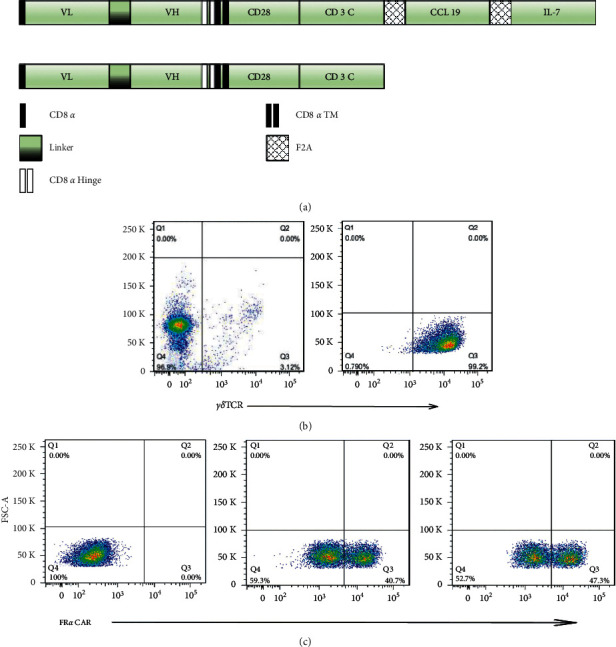
*γδ* T cells were successfully separated and expanded from healthy donor PBMCs and transduced with CAR structure. (a) Schematic representation of 7 × 19 CAR and conventional CAR constructs. (b) The cells were detected by flow cytometry and representative dot plot from the same donor showing the percentage of V*γ*9V*δ*2 cells in PBMCs (left) and after the MACS magnetic beads sorting (right). (C) FACS analysis of sorting *γδ*T cells transduction efficiency. The sorted *γδ*T were stimulated with ZOL, IL-2, and IL-21 and transfected with either 7 × 19 CAR (middle) or conventional CAR (right). The untransduced *γδ*T were treated as negative control.

The 7 × 19 CAR construct was comprised of the Mov19 scFv linked to a CD8a hinge and transmembrane region, CD28 intracellular signaling motif, and CD3z signaling moiety tandems in proper order, followed by the genes encoding two 2A peptide sequence between IL-7 and CCL19 (Figure 1(a)). The conventional CAR construct containing the same elements except for the absence of IL-7 and CCL19 was generated as control (Figure 1(b)). The amino acid sequence of 7 × 19 CAR and conventional CAR was cloned in CAR trans-gene plasmids, respectively, for future use.

### 2.3. Lentivirus Production

High-titer replication-defective lentiviruses were produced and concentrated according to the manufacturer's instructions. Briefly, 293T cells were seeded in 150-cm^2^ flask. Either 7 × 19 CAR trans-gene plasmids or conventional CAR trans-gene plasmids (15 *μ*g) were cotransfected with 18 *μ*g pRSV.REV, 18 *μ*g pMDLg/*p*.RRE, and 7 *μ*g pVSV-G with 174 *μ*L lipo2000 (1 *μ*g/*μ*L) per flask. Supernatants were collected at 48 h after transfection and concentrated 10-fold by ultracentrifugation for 2 h at 28000 rpm with a Beckman SW32Ti rotor. Viruses were aliquoted into tubes and stored at −80°C until ready to use for titering or experiments.

### 2.4. Preparation of Human *γδ*T Cells

Peripheral blood samples were obtained from healthy donors at Hebei Blood Center, Hebei, China. Informed consents were obtained from all donors in accordance with the guidelines approved by Hebei Medical University, Hebei, China. Peripheral blood mononuclear cells (PBMCs) were isolated by Ficoll-Paque density gradient centrifugation and collected from the interphase. After washing with PBS for three times, the PBMCs were cultured in RPMI-1640 complete medium supplemented with 10%FBS in adherent flask for 2–4 h. Then, the nonadherent cell was removed for sorting the positive *γδ*T cells by using human *γδ*T cell isolation MicroBeads Kit (Miltenyi Biotec, Germany). The sorting processes were operated according to the manufacturer's instructions, and then, the *γδ*T positive T cells were cultured and stimulated in RPMI-1640 complete medium supplemented with 10%FBS (R10), 300U/ml recombinant human IL-2 (R10 medium), 5 *μ*M zoledronic acid, and 10 ng/mL IL-15 (Life Technologies, Rockville, MD, USA). *αβ*T cell were purified from PBMCs following the same procedure by using human CD [3]+T cell isolation MicroBeads Kit, and then, the *αβ*T cells were activated with OKT3/CD28 and cultured in R10 medium for future use.

### 2.5. Generation of 7 × 19 CAR-*γδ*t*γδ* and CAR-*γδ*t*γδ* Cells

Approximately 24 h after activation, human T cells were transduced using a spinoculation procedure. Briefly, T cells were infected with a multiplicity of infection (MOI) of 5 of the 7 × 19 CAR lentivirus vector. A mixture of cells and vectors were centrifuged at room temperature for 90 min (2500 rpm) in a table-top centrifuge (Sorvall ST 40). Then, rested T cells were used for functional analysis.

### 2.6. Cell Migration Assay

Chemotaxis of the B cells, *αβ*T cells, or DCs was measured by migration through a polycarbonate filter of 5-µm pore-size in 96-well transwell chambers (Millipore, Billerica, MA, USA). The process was performed according to the manufacturer's instruction. Briefly, 7 × 19 CAR-*γδ*T, CAR-*γδ*T or *γδ*T cells were stimulated in the lower chambers, and the responder cells, including PBMCs, CFSE-labeled *αβ*T cells, or CFSE-labeled DCs, were incubated in the upper chambers for 4 h. The cells that migrated from the upper chamber to the lower chamber were assessed and counted by flow cytometry.

### 2.7. Flow Cytometry Analysis

To demonstrate positive CAR expression and antigen binding capacity, T cells surface CAR expression was evaluated using recombinant FR*α*-Fc protein followed by PE-labeled anti-human IgG Fc gamma-specific antibody. The T cell phenotypic were measured by PE-anti-human TCR*γ*/*δ*. Expression of CCR7 in human PBMCs, *αβ*T cells, and DC cells was detected by APC-anti-human CCR7 antibody. Human breast cancer cell line MCF-7, human TNBC tumor cell line MDA-MB-231, MDA-MB-436, MDA-MB-468, and human ovarian cancer cell line C30 cell line surface expression of FR*α* were measured using human PE-FR*α* mAb. B cell migration was identified by using the same gate as in PBMC.

All flow cytometry was conducted using a BD FACS Canto flow cytometer (BD Biosciences), and flow cytometric data were analyzed with FlowJo version 7.6.1 software.

### 2.8. In Vitro Stimulation of CAR-T Cells

For stimulation of FRa specific 7 × 19 CAR-T and conventional CAR-T cells, MDA-231 cells were inactivated with 50ug/ml mitomycin C for 90 min at 37 °C. Cytokine release assays were performed by coculturing 7 × 19 CAR *T*, conventional CAR *T*, or UNT cells with 1 × 10^5^ MDA-231 or C30 cells in triplicate in a 96-well flat bottom plate in a total volume of 200 *μ*L. After 20–24 h, cocultured supernatants were collected and ELISA (Biolegend, San Diego) was performed, according to the manufacturer's instructions, to measure the IFN-*γ*, IL-7, and CCL19, which were included in the culture medium. The values showed represent the mean of triplicate wells.

### 2.9. Cytotoxicity Assays

For cytotoxicity assay, 5 × 10 [4] MCF-7, MDA-231, or C30 cells were cultured with RPMI-1640 complete media in the presence of different effector cells ratios in 96-well Microplate. After coculturing for 12 h, tumor cell viability percentage were measured by CCK-8 kit through exhibiting the absorbance at 450 nm by a microplate reader. Cytotoxicity results were calculated using the following formula: specific lysis (%) = [1– (mixture cell experiment–medium control)/(target cell spontaneous–medium control)]×100%. All data are represented as a mean of triplicate wells.

### 2.10. Treatment of Breast Cancer or TNBC Xenograft Model

To evaluate the antitumor ability of 7 × 19 CAR-T in vivo, six-to-eight-week old, female nonobese diabetic/severe combined immunodeficient (NOD/SCID) mice were purchased from Charles River Laboratories (Beijing. China) and bred, treated, and maintained under specific pathogen-free (SPF) condition. Mice were inoculated subcutaneously on the right flank with either 3 × 10 [6] MDA-231 cells/0.3 ml PBS or 3 × 10 [6] MCF-7 cells/0.3 ml PBS on day 0. After tumor volume reached 150–200 mm [3], the MCF-7 cells bearing mice were randomly divided into four groups and treated with 2 × 10 [7] PBMC/0.2 ml PBS, 2 × 10 [7] *γδ*T/0.2 ml PBS, 2 × 10 [7] CAR-*γδ*T/0.2 ml PBS, and 2 × 10 [7] 7 × 19 CAR-*γδ T*/0.2 ml PBS, respectively. The MDA-231 cells bearing mice were randomly divided into six groups and treated with 2 × 10 [7] PBMC/0.2 ml PBS, 2 × 10 [7] *γδ*T/0.2 ml PBS, 2 × 10 [7] CAR-*γδ*T/0.2 ml PBS, 2 × 10 [7] 7 × 19 CAR-*γδ*-T/0.2 ml PBS, 2 × 10 [7] CAR-*γδ*T+ 2 × 10 [7] PBMC/0.2 ml PBS, and 2 × 10 [7] 7 × 19 CAR-*γδ* T+ 2 × 10 [7] PBMC/0.2 ml PBS through tail vein injection, respectively. The tumor size was measured using caliper twice a week, and the tumor volumes were calculated using formula V = (length × width (2))/2. When the tumor volumes reach nearly 1500 mm^3^, the mice were sacrificed, and tumor samples were resected to calculate the final volume. The mice were euthanized with carbon dioxide. The euthanasia box does not contain carbon dioxide before placing the animal. After placing the animals, fill the euthanasia box with CO_2_ at a rate of 10% of the replacement box volume per minute. After 10 min, the mice did not breathe, and their pupil dilated. The mice were observed for another 2 min and confirmed to be completely dead.

### 2.11. Histopathological and Immunofluorescence Assays

After the TNBC xenograft tumor was resected, samples were stored in paraformaldehyde fixative and then embedded in paraffin. For hematoxylin and eosin (H&E) staining, paraffin sections were first dewaxed and stained with hematoxylin. Sections were then dehydrated in an ethanol gradient and stained with eosin. In immunofluorescence (IF) analysis, anti-CD3 antibody and anti-S100 antibody were used as primary antibody and nuclei were stained with DAPI. Representative pictures from three independent experiments are shown.

### 2.12. Statistical Analysis

The data are reported as means and standard deviations (SDs). Statistical analysis was performed using two-way repeated-measure analysis of variance (ANOVA) for the tumor volumes. Student's *t*-test was used to evaluate differences in absolute numbers of transferred T cells, cytokine secretion, and specific cytolysis. GraphPad Prism 5.0 (GraphPad Software) was used for the statistical calculations, where a *p* value of *p* < 0.05 was considered significant.

## 3. Results

### 3.1. Construction and Expression of Either FR*α*-Specific 7 × 19 CAR or Conventional CAR in *γδ*T Cells from Peripheral Blood

The percentage of *γδ*T in the peripheral blood varies from 3%–10% (Figure 1(b), left). Through magnetic bead negative sorting, the enriched cells were amplified by stimulation of zoledronic acid, IL-2, and IL-15, and the concentration could reach 99% (Figure1(b), right). The FR*α* targeting CAR-encoding lentivirus construct was generated by fusing the MOv19 scFv, CD8*α* hinge and transmembrane region, CD28 intracellular signaling motif, CD3*ζ* signaling moiety, two 2A peptide sequences to separate the conventional CAR structure, IL-7, and CCL19. Two CAR structures were named as 7 × 19 CAR or conventional CAR (Figure 1(a)). Purified *γδ*T cells were efficiently transduced with either 7 × 19 CAR or CAR lentiviral vectors with the transduction efficiency of nearly 45% (Figure 1(c)).

### 3.2. CCR7 Is an Attractive Chemokine Receptor in Various Immune Cells

**Figure 2:**
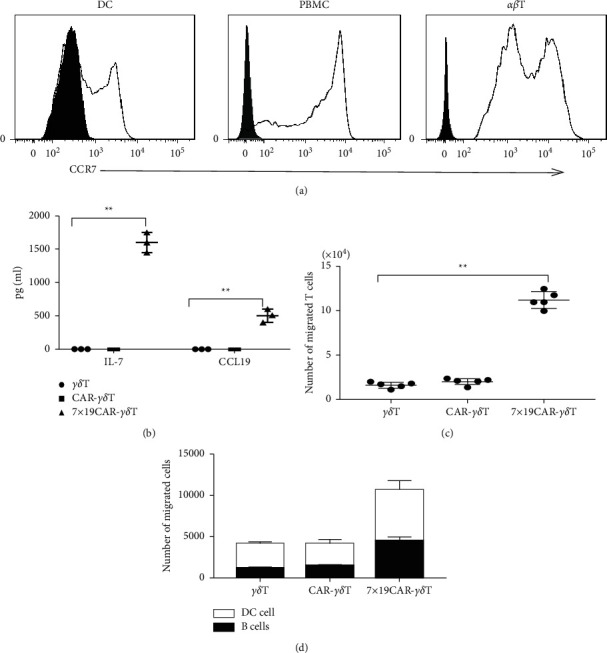
Chemotactic capacity towards *γδ*T, CAR-*γδ*T, and 7 × 19 CAR-*γδ* T cells. (a) FACS analysis of the expression of CCR7 in human dendritic cells (left), PBMCs (middle), and *αβ* T cells (right). (b) IL-7/CCL19 secretion of *γδ*T, CAR-*γδ*T, and 7 × 19 CAR-*γδ*T were detected by ELISA, respectively (*p* ＜ 0.01). (c) The purified *αβ* T cell migrated from the upper chamber to the lower chamber and was analyzed by flow cytometry (*p* ＜ 0.01). (d) B cells and CFSE-labeled dendritic cells migration driven by CXCL13 and CCL19 were counted using flow cytometry. *P* values were calculated by Student's two-sided *t*-test.

The chemokine CCL19 and its receptor CCR7 have a wide range of sources in the body. CCL19/CCR7 is involved in the homing of immune cells and the occurrence of inflammatory response in the body. The flow cytometry results indicated that 50% of the DC cells, the majority of PBMC, and the 100 percent of *αβ* T cells are CCR7 positive expression (Figure 2(a)). The bimodal distribution of *αβ T* indicates different fluorescence intensities, which reveals the different expression level of CCR-7 in CD4/CD8 T cells (Figure 2(a), right). The secretion of CCL19 and IL-7 was then measured using ELISA. The results show that the activated 7 × 19 CAR-*γδ T* can secrete CCL19 and IL-7 exclusively, while the CAR-*γδ T* or *γδ T* cannot (Figure 2(b)). Then, we performed transwell migration assays. The migration of responder T cells, DC cells, and B cells was significantly enhanced by incubation with 7 × 19 CAR-T cells compared with conventional CAR-T cells (Figures 2(c) and 2(d)).

### 3.3. FR*α* Is Expressed on the Surface of TNBC Cell Lines Moderately

**Figure 3:**
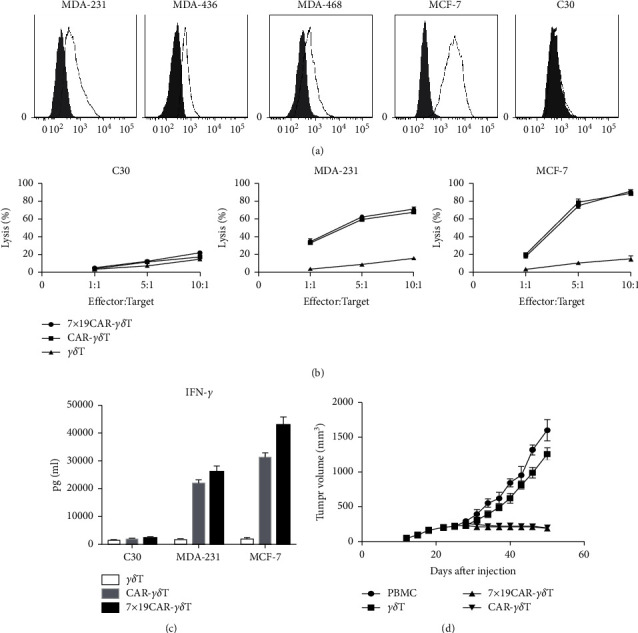
Antitumor ability of *γδ*T, CAR-*γδ*T, and 7 × 19 CAR-*γδ* T cells both in vitro and in MCF-7 CDX model. (a) FACS analysis of the expression level of FRa in human TNBC cell line MDA-231, MDA-436, MDA-468, human breast cancer cell line MCF-7, and human ovarian cancer cell line C30. (b) FRa-specific cytotoxicity curves of *γδ*T, CAR-*γδ*T, and 7 × 19 CAR-*γδ* T cells against FRa negative expression C30 (left), FRa moderate expression MDA-MB-231 (middle), and FRa overexpression MCF-7 (right) tumor cell lines at the ratio of 1 : 1, 5 : 1, 10 : 1, respectively. (c) Histograms showed IFN-*γ* secretion of *γδ*T, CAR-*γδ*T, and 7 × 19 CAR-*γδ*T against C30, MDA-MB-231, and MCF-7 at the ratio of 10 : 1. The IFN-*γ* production was detected using ELISA. (d) Tumor volume curves of FRa overexpression in MCF-7 CDX models. Quantification of tumor volumes (mm3) in the PBMC, *γδ*T, CAR-*γδ*T, and 7 × 19 CAR-*γδ* T treatment groups.

Using flow cytometry, surface expression of FR*α* protein was detected on human breast cancer cell line MCF-7, human TNBC tumor cell line MDA-MB-231, MDA-MB-436, and MDA-MB-468, and human ovarian cancer cell line C30 cell line after staining the cells with anti-FR*α* antibody. As shown in Figure 3. FR*α* is overexpressed in MCF-7 and is moderately expressed in MDA-MB-231, MDA-MB-436, and MDA-MB-468 but is negative in C30 cell line.

### 3.4. 7 × 19 CAR-*γγδ* T Cells Specifically Lysed FR*α*pos Tumor Cells In Vitro

CCK-8 assay was performed to detect the killing effects of 7 × 19 CAR-*γδ T* and conventional CAR-*γδ* T cells (effector cells). FR*α*^pos^ MCF-7 and MDA-231 cells were used as target cells, and FR^neg^ C30 were used as a negative control. 7 × 19 CAR-*γδ T* and conventional CAR-*γδ* T cells were cocultured with MCF-7, MDA-231, or C30 with different E : T ratios(1 : 1, 5 : 1, and 10 : 1), respectively. As shown in Figure 3(b), both of the 7 × 19 CAR-*γδ T* and conventional CAR-*γδ T* demonstrated strong cytotoxicity against FR*α*^pos^ MCF-7 and the killing efficiency was dose-dependent as the ratio of effector cells increased, but the untransduced *γδ T* did not show that tendency.

As a negative control, both of the 7 × 19 CAR-*γδ T* and conventional CAR-*γδ* T cells did not show obvious cytotoxicity when cocultured with FR*α*^neg^ C30 cells, and there was no statistically significant difference compared with untransduced *γδ* T cells. For the FR*α* moderate expressed MDA-231 cells, 7 × 19 CAR-*γδ T* and conventional CAR-*γδ T* can target the FR*α*^pos^ tumor cells, and the cytotoxicity was matched with the FR*α* expression level. Those results indicated that either 7 × 19 CAR-*γδ T* or conventional CAR-*γδ* T cells can specifically lyse tumor cells, where FRa are positively expressed.

### 3.5. 7 × 19 CAR-*γγδ T* Secrete Cytokines in Response to Tumor Stimulation

Cytokines IFN-*γ* in the effect cells against target cells in cocultured supernatants were detected by ELISA assay. As shown in Figure 3(c), a robust amount of IFN-*γ* was observed in either 7 × 19 CAR-*γδ*T or CAR-*γδ*T groups against MCF-7 or MDA-231. Moreover, the secretion of 7 × 19 CAR-*γδ*T was significantly higher than that of CAR-*γδ*T, which may be associated with the activated functions of IL-7. When 7 × 19 CAR-*γδ*T or conventional CAR-*γδ*T or *γδ*T were cocultured with C30 cells, there were no significant statistical difference among these groups. Those results demonstrated that 7 × 19 CAR-*γδ*T can activate costimulatory molecules through targeting and combining specific antigens and the cytotoxicity can be enhanced by IL-7.

### 3.6. 7 × 19 CAR-*γδ*t*γδ* Induced Tumor Regression In Vivo

**Figure 4:**
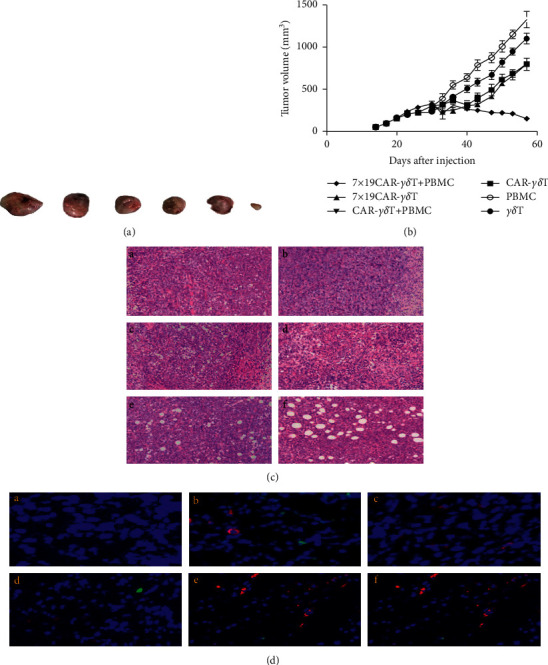
Antitumor ability of *γδ*T, CAR-*γδ*T, and 7 × 19 CAR-*γδ* T cells in FRa moderate expression MDA-MB-231 CDX model. (a) Tumor size comparison. Tumor xenografts were excised after treatment with different effector cells. One representative sample was displayed for each group. (b) Tumor volume curves of FRa overexpression MCF-7 CDX models. Quantification of tumor volumes (mm3) in the PBMC, *γδ*T, CAR-*γδ*T, 7 × 19 CAR-*γδ* T, and the combination of PBMC with either CAR-*γδ*T or 7 × 19 CAR-*γδ*T treatment groups. (c) H&E analysis of different treatment groups: (a–f) *γδ*T, PBMC, CAR-*γδ*T, 7 × 19 CAR-*γδ* t CAR-*γδ*T + PBMC, 7 × 19 CAR-*γδ* T + PBMC treatment groups, respectively (40×). (d) Immunofluorescence (IF) analysis of T cells and DC cells infiltration into the tumor xenografts. (A–F) *γδ*T, PBMC, CAR-*γδ*T, 7 × 19 CAR-*γδ* T CAR-*γδ*T + PBMC, and 7 × 19 CAR-*γδ* T + PBMC treatment groups, respectively (200×).

As 7 × 19 CAR-*γδ*T and CAR-*γδ*T can lyse FRa ^pos^ target cells in vitro specifically, we next evaluate its antitumor ability in vivo. In MCF-7 CDX model, the single use of 7 × 19 CAR-*γδ*T or CAR-*γδ*T can suppress tumor growth significantly compared with PBMC or *γδ*T treatment groups (Figure 3(d)). Then, we applied the 7 × 19 CAR-*γδ*T to find out its treating ability in the FRa moderate expression xenograft model. The mice were treated with certain numbers of effector cells through tail vein injection. Notably, 7 × 19 CAR-*γδ*T + PBMC treatment group is the only effective treatment group that suppressed tumor growth prominently compared with other groups (Figure 4(b)). IF analysis indicated that 7 × 19 CAR-*γδ*T + PBMC treatment groups induced massive T-cell infiltration in the tumor tissue, substantially higher than conventional CAR-*γδ*T + PBMC treatment groups. Furthermore, infiltration of DCs into the tumor tissues and their colocalization with T cells were observed after treatment with 7 × 19 CAR-*γδ*T + PBMC but not conventional CAR-*γδ*T + PBMC cells, indicating that 7 × 19 CAR-*γδ*T cells are capable of recruiting endogenous DCs into tumor tissues (Figures 4(d)–4(f)). The single use of the same does of CAR-*γδ*T suppressed tumor growth for a short time after the infusion, but then, the tumor grew rapidly, which may be related to the loss of antigens, and the absence of other immune cells plays a synergy role at the tumor site. The HE analysis also displayed large areas of vacuolar necrosis of the 7 × 19 CAR-*γδ*T + PBMC treated tumor tissues (Figures 4(c)–4(f)), while no extensive necrosis was observed in other groups.

## 4. Discussion

Serval factors contribute to the unavailability of effective treatment methods for TNBC. On the one hand, due to the scarcity of ideal targets, adoptive cell therapy such as CAR-T or TCR-T cells cannot specifically recognize and bind to the tumor cells. On the other hand, the presence of tumor microenvironment (TME) in the solid tumor had also limited the cytotoxicity of immune cells.

Song et al. [21] showed the remarkable killing efficiency in gene engineered FRa antigen overexpression xenograft model, indicating that FRa is a promising target in the treatment of patients whose FRa antigen are overexpressed. In natural TNBC cell-derived xenograft (CDX) model, the second-generation CAR-T cells targeting FRa did not show that tendency. This phenomenon can be regarded as sequential antigen loss [22, 23], and it is reasonable that the FRa-CAR *T* can lyse the Fra-positive tumor cells specifically, but not the residual Fra-negative tumor cells, which leads to the ineffectiveness of FRa-targeting cell therapy.

*γδ*T cells can target the tumor cells through non-MHC restricted mode naturally [24]. After the target cells were lysed, tumor antigens were took up and processed as associated peptide by *γδ*T cells and then presented to the *αβ*T cells [25, 26]. *γδ*T cells can also secrete CXCL13, which regulated the migration of B cells. It may motivate the body's innate humoral immunity to exert the antitumor response [9]. Some studies have pointed that the lifespan of *γδ*T cells is shorter than that of *αβ*T cells [27], which is possibly due to their inability to colonize bone marrow. This may also reduce the long-term toxicity of CAR-T to a certain extent, although it has an adverse effect on the maintenance of the body's long-term antitumor response [28]. In addition, 7 × 19 CAR-*γδ*T infusion can reduce the adverse effects of CAR-T-associated side effects by reducing the dose of cells in a single infusion while maintaining the long duration of the antitumor immune response by playing the role of APC and promoting the formation of memory associated phenotype antitumor T cells [29, 30].

In this study, we introduced chemokines CCL19 and IL-7 into the structure of conventional CAR, each domain was separated using 2A ligating peptides to ensure their respective functions. Either the FRa-targeting CAR-*γδ*T or 7 × 19 CAR-*γδ*T suppressed the FRa overexpressed MCF-7 tumor cells both in vitro and in vivo. But for the FRa moderately expressed MDA-MB-231 cells, CAR-*γδ*T or 7 × 19 CAR-*γδ*T can lyse the Fra-positive cells in vitro and cannot inhibit tumor growth in MDA-MB-231 CDX model when applied separately. The IHC assay found that CAR-*γδ*T and 7 × 19CAR-*γδ*T can hardly infiltrate to the tumor tissues. Then, we applied the combination of 7 × 19 CAR-*γδ*T cells and PBMCs in the treatment of MDA-MB-231 CDX model. Intriguingly, after the coinfusion of 7 × 19 CAR-*γδ*T cells and PBMCs, the tumor growth was significantly inhibited and IHC indicated that plenty of DCs and CD3 positive cells infiltrated to the tumor tissues compared with other groups.

In summary, our study presented that 7 × 19 CAR-*γδ*T can suppress natural TNBC CDX model by lysis of the FRa positive cells and secreting CCL19 and IL-7. CCL19 can make chemotactic DC cells and *αβ*T cells infiltrate into tumor tissue, and those *αβ*T cells can be activated by the APC cells such as DC cells and *γδ*T cells and played a synergistic role in fighting against the FRa-negative TNBC cells. The IL-7 enhanced the proliferation and survival ability of T cells that infiltrated into tumor tissues, which reduced the inhibitory of tumor microenvironment [30]. These results provide insights into the application of universal 7 × 19 CAR-*γδ*T in solid tumor treatment and explored a new method to enhance the ACT antitumor ability by mobilizing the normal immune cells.

## 5. Conclusion

Our findings demonstrated that 7 × 19 CAR-*γδ*T can not only lyse the target tumor cells, but also break through the TME and lyse nontarget tumor cells through motivating the body's own immunity.

## Data Availability

The analyzed datasets generated during the study are available from the corresponding author on reasonable request.
